# A Novel Tropically Stable Oral Amphotericin B Formulation (iCo-010) Exhibits Efficacy against Visceral Leishmaniasis in a Murine Model

**DOI:** 10.1371/journal.pntd.0000913

**Published:** 2010-12-07

**Authors:** Ellen K. Wasan, Pavel Gershkovich, Jinying Zhao, Xiaohua Zhu, Karl Werbovetz, Richard R. Tidwell, John G. Clement, Sheila J. Thornton, Kishor M. Wasan

**Affiliations:** 1 School of Health Sciences, British Columbia Institute of Technology, Burnaby, Canada; 2 Faculty of Pharmaceutical Sciences, University of British Columbia, Vancouver, Canada; 3 Division of Medicinal Chemistry and Pharmacognosy, College of Pharmacy, The Ohio State University, Columbus, Ohio, United States of America; 4 Department of Pathology and Lab Medicine, Consortium for Parasitic Drug Development, University of North Carolina, Chapel Hill, North Carolina, United States of America; 5 iCo Therapeutics Inc., Vancouver, Canada; Hebrew University, Israel

## Abstract

**Purpose:**

To develop an oral formulation of amphotericin B (AmB) that is stable at the temperatures of WHO Climatic Zones 3 and 4 (30–43°C) and to evaluate its efficacy in a murine model of visceral leishmaniasis (VL).

**Methods:**

The stability testing of four novel oral lipid AmB formulations composed of mono- and di-glycerides and pegylated esters (iCo-010 to iCo-013) was performed over 60 d and analyzed by HPLC-UV. In addition, the four formulations were incubated 4 h in fasted-state simulated intestinal fluid. AmB concentration was measured spectrophotometrically and emulsion droplet diameter was assessed by dynamic light scattering. Antileishmanial activity of iCo-010 was evaluated at increasing oral doses (2.5 to 10 mg/kg) in a murine model of VL.

**Results:**

AmB stability in the lipid formulation (iCo-010) was >75% over 60 days. After 4 h in fasted-state simulated intestinal fluid, AmB concentration was >95%. iCo-010 demonstrated significant efficacy when orally administered to VL-infected mice bid for five days (inhibition of 99%, 98%, and 83% at 10, 5 and 2.5 mg/kg compared to the vehicle control). In addition, the qd dose of 20 mg/kg provided 96% inhibition compared to the vehicle control.

**Conclusions:**

The oral AmB formulation iCo-010 is stable at the temperatures of WHO Climatic Zones 3 and 4 (30–43°C). iCo-010 showed excellent antileishmanial activity at both 10 mg/kg po bid for 5 days (<99% reduction in parasitic infection) and 20 mg/kg po qd for 5 days (95% inhibition when compared to control).

## Author Summary

Visceral leishmaniasis (VL) is a systemic form of a vector-borne parasitic disease caused by obligate intra-macrophage protozoa of the genus *Leishmania*. VL is always fatal in humans if left untreated and treatment options are limited. Amphotericin B (AmB), a polyene antibiotic, is the most active antileishmanial agent that currently exists. Liposomal AmB (AmBisome) is used as first-line treatment in developed countries [1], [7], [8], [9], [10]; however, the requisite parenteral administration and the high cost of the liposomal formulation prevents this treatment from reaching the majority of patients in developing nations [3]. A stable, efficacious oral treatment for VL that is able to withstand the rigors of tropical climates would overcome many of the current barriers to treatment that exist in countries with large VL-infected patient populations. In this study we have developed an oral formulation of AmB that is stable in tropical conditions and exhibits significant antileshimanial activity in mice.

## Introduction

Visceral leishmaniasis (VL) is a systemic form of a vector-borne parasitic disease caused by obligate intra-macrophage protozoa of the genus *Leishmania*. VL is transmitted via the bite of an infected sand fly [1], [2]. The parasites are then disseminated through the vascular and lymphatic systems, infecting monocytes and macrophages of the reticulo-endothelial system and accumulating in the liver and spleen [1].

VL is always fatal in humans if left untreated [3]. Unfortunately, the treatment options for VL are limited. Amphotericin B (AmB), a polyene antibiotic, is the most active antileishmanial agent that currently exists. Liposomal AmB (AmBisome) is used as first-line treatment in developed countries [1], [7], [8], [9], [10]; however, the requisite parenteral administration and the high cost of the liposomal formulation prevents this treatment from reaching the majority of patients in developing nations [3]. A stable, efficacious oral treatment for VL that is able to withstand the rigors of tropical climates would overcome many of the current barriers to treatment that exist in countries with large VL-infected patient populations. An oral lipid-based formulation of AmB (iCo-009) was recently developed and evaluated in animal models of systemic fungal infections and VL [4], [5], [6]. iCo-009 demonstrated significant antifungal and antiparasitic activity as well as an excellent drug stability profile at 4°C [4], [5], [6].

The goal of the current study was to develop potential oral formulations of AmB that are a) stable at the temperatures of WHO Climatic Zones 3 and 4 (30–43°C); b) retain AmB stability in simulated gastric and intestinal fluids and c) exhibit significant antileishmanial activity in a VL-infected murine model. We have now developed a number of lipid-based self-emulsifying formulations which exhibit AmB chemical stability in FaSSIF and temperature stability at 43°C. The temperature stability of AmB at 30 and 43°C in a series of lipid formulations (iCo-010-013) are described, which indicate that formulations based on mono- and di-glycerides and Vitamin E-TPGS provide excellent temperature stability for AmB. Furthermore, these formulations show good stability in simulated intestinal fluids, which mimic the degradative stress of oral administration. A nanoemulsion (with droplet diameters <1 µm) forms in these fluids upon mixing at 37°C, facilitating absorption of these AmB-lipid compositions. In addition, we provide data that demonstrate the significant antileishmanial activity of one of these tropically stable, novel lipid-based oral AmB formulations (iCo-010).

## Materials and Methods

### Ethics Statement

Studies in Leishmania donovani-infected BALB/c mice were carried out according to a protocol approved by The Ohio State University Institutional Animal Care and Use Committee (National Institutes of Health Office of Laboratory Animal Welfare Assurance Number A3261-01). The study was conducted adhering to the Ohio State University's guidelines for animal husbandry and appropriate steps were taken to avoid or minimize pain and suffering.

### Materials

Amphotericin B (AmB; 80% purity) was purchased from Sigma Chemical Co. (St. Louis, MO) and used as received. Ethanol (100%) was purchased from Commercial Alcohols (Vancouver, BC). Mono- and di-glycerides were a gift from Gattefossé Canada (Toronto, ON, Canada). D-alpha-tocopheryl polyethylene glycol succinate (Vitamin E-TPGS; NF grade) was bought from Eastman Chemical Co. (Kingsport, TN). Methanol, HPLC water, acetonitrile and acetic acid were purchased from Fisher Scientific (Ottawa, ON Canada) and were of HPLC grade. Sodium chloride, hydrochloric acid, sodium hydroxide, dibasic potassium phosphate, sodium taurocholate and porcine pancreatin were bought from Sigma Chemical Co. Egg phosphatidylcholine (lecithin) was purchased from Avanti Polar Lipids (Alabaster, AL).

### Analysis of AmB Concentration by HPLC

Chromatography conditions: The HPLC column was a BDS Hypersil C18, 5 µm, 250×4.6 mm (Thermo Scientific, Waltham, MA), with a C18 guard column from Phenomenex (Torrance, CA). During sample runs, the column incubator was set to 40°C. The mobile phase consisted of acetonitrile: acetic acid: water in a ratio of 57∶4.3∶38.7 (v/v/v). The injection volume was 90 µL and the flow rate was 0.8 mL/min with a run time of 20 min. The retention time of AmB under these conditions was 15 min. Six triplicate standards of AmB in methanol: water (50∶50 v/v) were used for external calibration, with a linear range of 31.25–1000 ng/mL by linear regression analysis (r^2^>0.999). For analysis of AmB in lipid formulations, the samples were warmed in a 48°C waterbath to melt the lipids, followed by vigorous vortexing for 2 min. A double dilution was then used. With a micropipettor, 100 µL were transferred to a glass vial followed by the addition of 9.9 mL methanol and vortexing for 1 min. From this dilution, 100 µL was aliquoted to another glass vial, followed by addition of 9.9 mL of methanol:water (50∶50 v/v) and vortexed for 1 min, thereby making 1∶10,000 dilutions of the original samples. These final dilutions were analyzed by HPLC as described above and compared to the standard curve for quantification of AmB concentration.

### Formulation Stability at 30°C and 43°C

The stability testing of AmB in each of the four oral lipid formulations (AmB in mono- and di-glycerides with or without Vitamin E-TPGS; iCo 010-013) was performed at 30°C and 43°C at ambient humidity (>85%). The base compositions of the formulations are composed of mono- and diglycerides with a mixture of glycerol and PEG1500 esters of long fatty acids with or without Vitamin E-TPGS. iCo 010 contains 60/40 (v/v) mono- and diglycerides with Vitamin E-TPGS; iCo 011 contains 50/50 (v/v) mono- and diglycerides with Vitamin E-TPGS; iCo 012 contains 50/50 (v/v) mono- and diglycerides without Vitamin E-TPGS; and iCo 013 contains 60/40 (v/v) mono- and diglycerides without Vitamin E-TPGS. For stability analysis at 30°C and 43°C, multiple time points covering a 60 day period were chosen as part of the accelerated temperature studies set out by the WHO to mimic tropical climate Zone 3 and 4 conditions. Four independent replicate samples were prepared for each time point by melting the batch of AmB in lipids at 48°C and stirring to homogeneity, followed by quickly aliquoting 0.5 mL of the AmpB/lipid mixture into individual polypropylene microcentrifuge tubes. The tubes were then sealed with parafilm. Samples were protected from light with foil and stored in incubators at 30° or 48°C. The starting concentration of AmB in the lipids was measured by HPLC (3–4 mg/mL depending on batch concentration). All subsequent measurements of AmB during the course of the stability study were performed by HPLC as described above and are reported as “% of original concentration” as measured on the day the sample was aliquoted (day 0). The rate of loss of AmB per day was calculated as: ([AmB]_day 0_−[AmB]_day 60_)/60 days.  = µg/mL⋅day loss. These data are reported as mean ±SD for 4 independent replicates. Statistical differences were assessed pair-wise by Student's t test (paired, 2-tailed) (SigmaStat v.3.5) with significance set at *p*<0.05.

### Stability in Simulated Fasted-State Intestinal Fluid (FaSSIF)

Preparation of Fasted-State Simulated Intestinal Fluid without enzymes was also prepared according to the USP V.28 and was composed of 3.9 g/L dibasic potassium phosphate, 1.613 g/L sodium taurocholate ( = 3 mM), lecithin (egg phosphatidylcholine) 0.57 g/L ( = 0.75 mM), potassium chloride 7.7 g/L dissolved in water and with sufficient hydrochloric acid to adjust the pH to 6.5. AmB formulations in mono- and di-glycerides with or without Vitamin E-TPGS and the corresponding drug-free vehicle controls were melted at 48°C in a water bath followed by vigorous vortexing for 2 min. FaSSIF was warmed to 37°C in 3 foil-wrapped 500 mL beakers on stirring hotplates. For incubation in FaSSIF, samples (0.5 mL) were added to 249.5 mL FaSSIF to achieve a dilution of 1∶500 v/v. The samples were mixed vigorously at 37°C over over 4 h for FaSSIF incubation, producing an emulsified mixture. At 0, 10, 30, 45, 60, 90, 120 and 240 min in FaSSIF, triplicate 1 mL samples were withdrawn from the beakers while the mixing continued. The 1 mL samples were diluted with 9 mL methanol and vortexed to clarity. Quantification of AmB concentration was performed by ultraviolet spectroscopy (λ = 407) on a Carey Bio-300 UV-visible spectrophotometer (Varian Canada, Mississauga, Ontario) by comparison to an external standard curve consisting of UV measurements of AmB in methanol containing the same dilution of the corresponding vehicle control for each formulation type. The linear range by regression analysis was 0.5–1.75 µg/mL (r^2^>0.999).

### Emulsion Droplet Sizing in Simulated Fasted-State Intestinal Fluid

The translucent emulsion formed during incubation of AmB in oral lipids in simulated gastric or intestinal fluid tends to cream (oil to the top of the aqueous phase) if not continuously mixed. Therefore, 1 mL samples were obtained mid-beaker depth at 2 h and 4 h for FaSSIF incubations during vigorous mixing and transferred to a plastic cuvette. Samples were mixed again by vortexing prior to placement in the sizing instrument. The emulsion droplet size was measured by dynamic light scattering (Malvern Zetasizer, Malvern Instruments, Worchestershire, UK) operating with an argon laser (λ = 633 nm) and with the sample holder kept at 37°C for 3 runs of 1 minute each, during which time the measurements were stable. Intensity weighting was used. Data are reported as the mean droplet diameter ± standard deviation (SD) from mean of 3 separate samples, where three runs were averaged for each sample.

### *In vivo* evaluation of iCo-010 in a VL-infected Murine Model

To determine the antileishmanial activity of our oral AmB formulation, the following studies were completed. BALB/c mice were intravenously infected with 5×10^7^ *Leishmania donovani* LV82 promastigotes (obtained by culturing amastigotes taken directly from the spleen of an infected hamster) seven days prior to treatment. Following the seven days, mice were either administered five daily doses of miltefosine at 3 mg/kg po, or iCo-010 at 2.5, 5, and 10 mg/kg po bid for five consecutive days or 20 mg/kg po qd for five consecutive days. Appropriate vehicle controls were also assessed. Animals were sacrificed 14 days post infection and Leishman-Donovan units (LDU) were assessed in livers of mice post mortem via microscopic enumeration of Giemsa-stained liver smears.

## Results

### Formulation Stability

**Figure 1:**
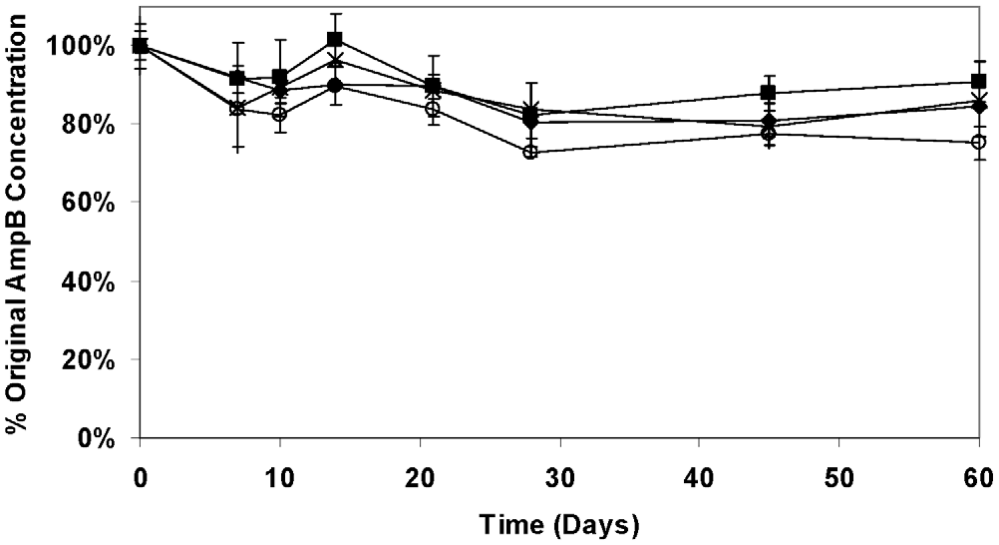
Stability of AmB in lipid suspensions at 30°C over 60 days. Symbols: solid diamonds: iCo-010; solid squares: iCo-011; crosses: iCo-012; open circles: iCo-013. Data represent mean ± SD (n = 4).

**Figure 2:**
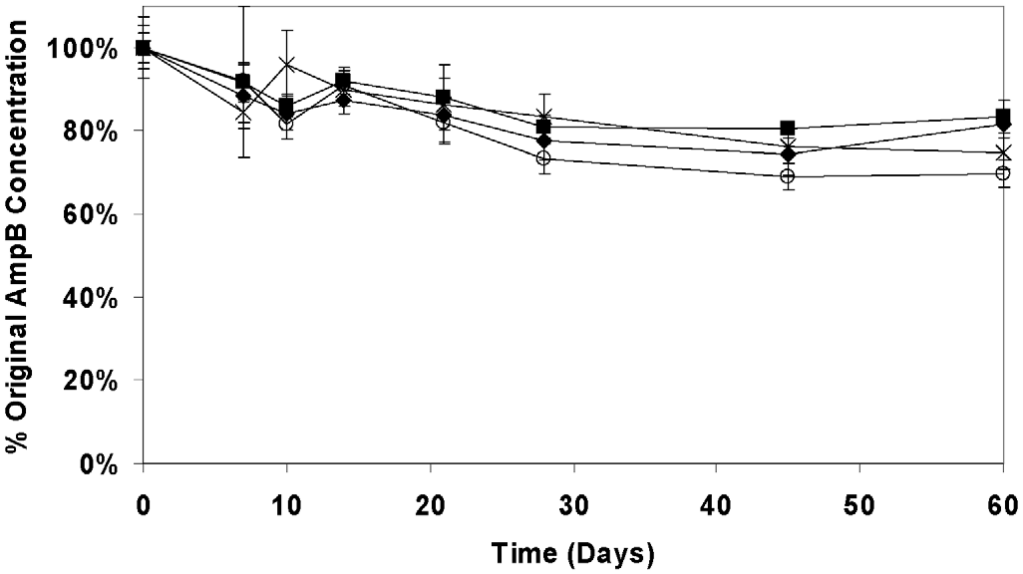
Stability of AmB in lipid suspensions at 43°C over 60 days. Symbols: solid diamonds: iCo-010; solid squares: iCo-011; crosses: iCo-012; open circles: iCo-013. Data represent mean ± SD (n = 4).

The results in **Fig. 1** show that the stability of AmB in the lipid formulations is >75% for all formulations over 60 days at 30°C and **Fig. 2** shows a similar pattern at 43°C with a slightly lower concentration in all formulations by 60 days. After 60 days at 30°C, AmB concentrations were comparable with and without Vitamin E-TPGS (∼85% of the original concentration), but when different proportions of mono- and di-glycerides were employed, samples without Vitamin E-TPGS (iCo-013) contained only 75% of the original AmB concentration compared to 91% when Vitamin E-TPGS was included (iCo-011). The addition of Vitamin E-TPGS did not significantly change the decomposition rate for AmB in iCo-010 at 43°C, although there was a trend to greater retention of AmB when Vitamin E-TPGS was included. The rate of decomposition (µg/mL. day) of AmB in all the lipid formulations at both temperatures is slow, as indicated in **Table 1**. There was a significant decrease (*p*<0.01) in the rate at which the AmB was lost in iCo-011 compared to iCo-013. This resulted in AmB concentrations after 60 days at 43°C that were 83.4% of the original concentration when Vitamin E-TPGS was included but only 69.7% when it was not present. It should be noted that no phase separation of the lipids or changes in particle size were observed after 60 days of incubation at 43°C for iCo 010.

**Table 1:** Decomposition rate of AmpB in lipid formulations.

Lipid formulation	30°C	43°C
	*µg/mL per day lost*	*µg/mL per day lost*
iCo-010	13.78±4.49	16.32±7.25
iCo-011	7.55±5.75	13.66±2.07
iCo-012	12.11±9.76	21.39±5.08
iCo-013	18.63±5.73	25.85±2.34

Data indicate mean rate of loss of AmB ± SD (n = 4). Results derived from the data in Fig. 1 and 2.

### Stability in Simulated Fasted-State Intestinal Fluid

**Figure 3:**
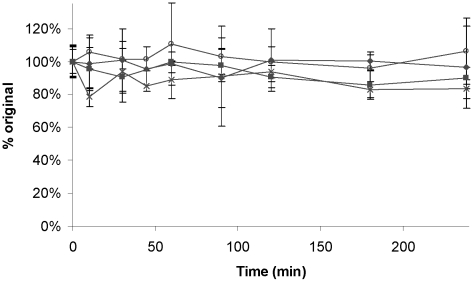
Stability of AmB in lipid formulations in fasted-state simulated intestinal fluid (FaSSIF) at 37°C. Symbols: solid diamonds: iCo-010; solid squares: iCo-011; crosses: iCo-012; open circles: iCo-013.Data represent mean ± SD (n = 3).

In simulated fasted-state intestinal fluid (FaSSIF), after 4 h of mixing at 37°C, iCo-010 retains >95% of its original drug concentration (**Fig. 3**). Assessment of the stability of AmB in iCo-011, iCo-012 and iCo-013 in FaSSIF shows very similar stability of formulations containing VitE-TPGS to those without it.

### Emulsion Droplet Sizing in Simulated Fasted-State Intestinal Fluid

**Figure 4:**
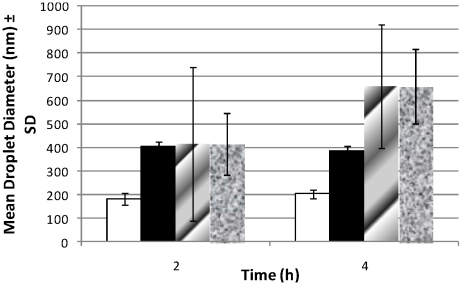
Emulsion droplets sizing in fasted-state simulated intestinal fluid. Symbols are white bars: iCo-010; black bars: iCo-011; dotted bars: iCo-012; hatched bars: iCo-013. Data represent mean ± SD (n = 3).

Assessment of emulsification in FaSSIF of AmB formulations composed of Peceol/Gelucire 44/14 shows that the presence of VitE-TPGS in the Peceol/Gelucire 44/14 mixture, such as in iCo-010 and iCo-011, reduces the mean diameter and creates a more monodisperse nanoemulsion. Emulsion droplet sizing of AmB in iCo-013 (no VitE-TPGS) following 2 and 4 h mixing in FaSSIF at 37°C revealed the largest droplet size of approximately 700–850 nm, whereas AmpB in iCo-010 (with VitE-TPGS) had a relatively smaller mean diameter closer to 200 nm. (**Fig 4**).

Considering the similar stability data for the four formulations tested (both at elevated temperature and in simulated gastrointestinal fluids) and more desirable self-emulsification properties of iCo-010, this formulation was chosen for *in vivo* studies of AmpB efficacy in an animal model of VL, as described below.

### Effectiveness in VL-infected Murine Model

**Figure 5:**
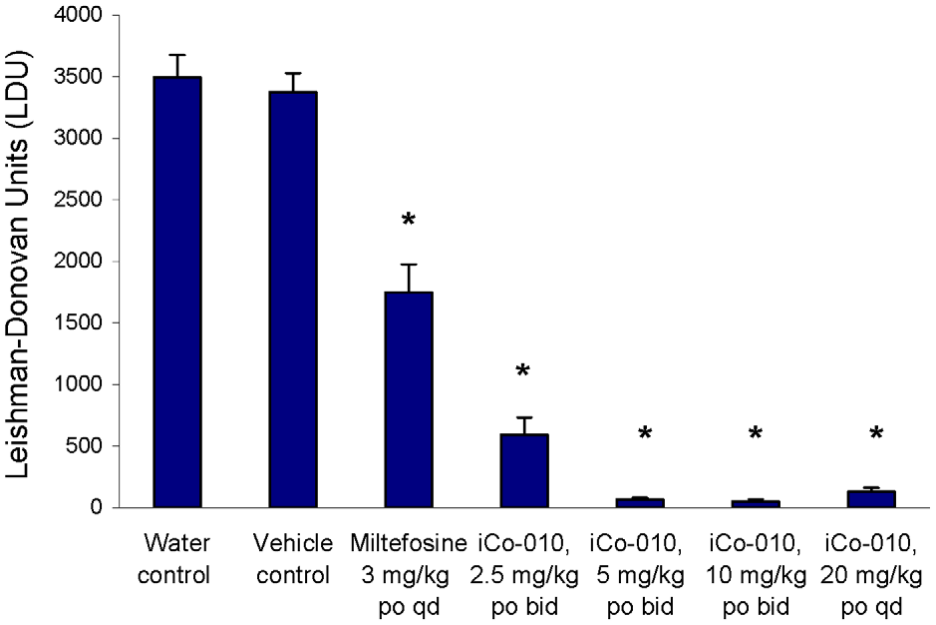
Antileishmanial activity of iCo-010 in *L. donovani*-infected BALB/c mice. Animals were infected and treated as described above, and LDUs were assessed by microscopic counting of liver smears. All treatments began seven days post infection. Bars of differing letters indicate statistically significant differences within each figure (one-way ANOVA with post-hoc Tukey Multiple Comparisons Test); data are expressed as mean ± SD. Groups of animals (n = 4) were treated with miltefosine at 3 mg/kg po daily for five days, a lipid-based vehicle control bid po for five days, 2.5, 5 and 10 mg/kg iCo-010 bid for five days or 20 mg/kg oral iCo-010 po qd for five days. Giemsa stained liver smears were obtained from mice post mortem after no treatment or exposure to vehicle, miltefosine, or the iCo-010 at the doses indicated. *: P<0.05 compared to vehicle control group. No significant difference between water control and vehicle control groups.

When given in five daily doses at 3 mg/kg po, miltefosine resulted in 47.5±7.0% inhibition of liver parasites, consistent with literature reports [6]. LDU values were not significantly different between groups of animals receiving oral doses of a lipid-based vehicle control bid for five days versus those receiving a single IV saline injection. (**Fig. 5**). Dose response data from treatment of *L. donovani-* infected BALB/c mice with 2.5, 5 and 10 mg/kg iCo-010 bid for five days is reported in **Fig. 5**. iCo-010 demonstrated significant efficacy when orally administered to VL-infected mice bid for five days (inhibition of 99%, 98%, and 83% at 10, 5 and 2.5 mg/kg compared to the vehicle control). In addition, the qd dose of 20 mg/kg provided 96% inhibition compared to the vehicle control. Based on data from our antifungal studies indicating no renal toxicity (data not shown) and the level of antileishmanial activity (**Fig. 5**), we are now confident that a self-administered tropically stable oral formulation of AmB is attainable.

## Discussion

The lipid formulations of AmB based on mono- and diglycerides ± Vitamin E-TPGS were developed as an alternative to the less temperature-stable monoglyceride formulation. Lipids with a melting point above ambient temperature and with good suspending properties, surfactant and self-emulsifying properties were chosen as alternatives. The ratio of mono- and di-glycerides and pegylated esters was selected based on preliminary studies of AmB temperature stability, component miscibility and maintenance of a stable suspension without phase separation. Furthermore, preliminary studies indicated that Vitamin E-TPGS in combination with mono- and di-glycerides and pegylated esters was tolerable in terms of maintaining a physically stable suspension of AmB at 5 mg/mL.

The temperature stability of AmB in all the lipid formulations was excellent, exceeding approximately 80% after 60 days at 30°C (**Fig. 1**) and 75% at 43°C (**Fig. 2**), and exhibiting no clear differences in the pattern of concentration loss *vs.* time amongst the four preparations. Upon examining the rate of drug loss as a function of excipient composition (**Table 1**), iCo-011 was slightly more stable than iCo-10 or iCo-013 at 30°C. As expected, addition of Vitamin E-TPGS significantly decreased the rate of drug loss at 43°C. At 43°C, the ratio of mono- and di-glycerides and pegylated esters appears less important than the presence of Vitamin E-TPGS, as formulations containing it showing a slower rate of AmB loss. However, the complex and likely temperature-dependent mechanism of interaction between AmB and the various lipids at the molecular level has not yet been explored. Nevertheless, these are very small differences and the biological properties of these formulations (bioavailability, tissue drug distribution and efficacy) would clearly be more important parameters to govern selection of a lead candidate from this set of formulations.

Regarding the properties of these lipid formulations in conditions mimicking those of the gastrointestinal tract, studies of the stability of the lipid formulations in FaSSIF demonstrate that AmB concentration is well-maintained when incorporated into all of the formulations, as shown in **Fig. 3**.

The self-emulsifying properties of these lipid formulations are deemed important because the formation of a nanoemulsion in the gastrointestinal tract may facilitate intestinal absorption, particularly through the lymphatic transport pathway. Furthermore, a major precipitation of the drug in gastrointestinal fluids would be undesirable and lead to unpredictable absorption patterns. The emulsification of the drug-lipid mixture also promotes interaction with bile salts, which can further enhance solubilization and absorption of the drug in the intestine. iCo-010 in FaSSIF, which contains sodium taurocholate as the bile salt, produced a slightly larger nanoemulsion of greater polydispersity. The emulsification behavior of the lipid formulations of AmB containing Vitamin E-TPGS in FaSSIF are an ongoing study which should allow for the further discernment of the role of Vitamin E-TPGS in the physical properties of these suspensions.

Considering the similar stability data for the 4 formulations tested (both at elevated temperature and in simulated gastrointestinal fluids) and more desirable self-emulsification properties of iCo-010, this formulation was chosen for in vivo studies of AmpB efficacy in an animal model of VL. The demonstrated efficacy of this formulation is likely due to a combination of increased solubility, improved gastrointestinal stability and enhanced membrane permeability. In addition, oral administration of a lipid-based formulation favors lymphatic transport. As VL parasites disseminate through the lymphatic and vascular system, infecting macrophages and infiltrating the bone marrow, liver and spleen, the lipid carrier may assist in delivering the drug to the site of greatest infection. Taking into consideration the short treatment course in this study, it is conceivable that longer treatment would completely eradicate VL in all treated animals. To the best of our knowledge, these data represent the first tropically stable oral AmB formulations to exhibit significant efficacy against *Leishmania donovani* (the parasite responsible for VL) in an infected mouse model.
